# Feasibility and comparison of left ventricular ejection flow acceleration recorded by cardiac magnetic resonance in patients with dilated cardiomyopathy: a case-control study

**DOI:** 10.1186/1532-429X-17-S1-P63

**Published:** 2015-02-03

**Authors:** Sébastian Tavolaro

**Affiliations:** 1 Radiology, European Hospital Georges Pompidou, Paris, France; 2 INSERM, Paris, France

## Background

Intra-ventricular pressure gradient along the direction of the outflow tract is generated by the impulse response of the myocardial contraction. This spatial pressure gradient, derived from the maximal acceleration estimates within flow along the left ventricular out flow tract (LVOT), has been validated in vitro by using MRI and was found strongly related to the inotropic state of the LV as demonstrated in animal and human echocardiographic studies. This acceleration may be less dependent on load conditions than others LV systolic function parameters, especially LV ejection fraction (LVEF). The aim of this study was to evaluate the feasibility of LV ejection flow acceleration estimated by spatial and temporal derivative of velocities recorded by cardiac magnetic resonance (CMR) using phase contrast sequence, and to compare the acceleration values to LVEF in patients with dilated cardiomyopathy (DCM) and in control subjects.

## Methods

Fourteen DCM patients and fourteen controls (mean age: 56 vs. 33 years old respectively) underwent CMR. Through plane phase contrast acquisitions were obtained in breathhold at the level of aortic valve and 10 mm below through the LVOT. Total acceleration (TotalAcc) and its two components, Temporal (TempAcc) and Convective (ConvAcc) acceleration were estimated from velocities derived with time and space obtained at the two different levels by using our home-made previously validated semi-automatic software Art-FUN.

## Results

Acceleration measurement was feasible in all controls and in fourteen out of seventeen patients (82%). TotalAcc and TempAcc (R^2^=0.45, p<0.0001 and R^2^=0.49, p<0.0001 respectively) but not ConvAcc (R^2^=0.004, p=0.74) were correlated with LVEF. Compared to control, mean LVEF (25±9 vs. 66±5, p<0.0001) TotalAcc (1097±317 vs. 1818±306 cm.s^-2^, p<0.0001) and TempAcc (1066±272 vs. 1656±257 cm.s^-2,^ p<0.0001) but not ConvAcc (706±478 vs. 840±613 cm.s^-2^, p=0.743) were significantly lower in DCM.

## Conclusions

This preliminary study have shown the feasibility of LV ejection flow acceleration measurement derived from velocities obtained 2 breatholding by using phase contrast sequence in CMR at two level perpendicular to the LVOT. TotalAcc and TempAcc are significantly lower in DCM patients compared to controls. TempAcc is correlated to LVEF, better than TotalAcc.

## Funding

N/A.

**Figure 1:**
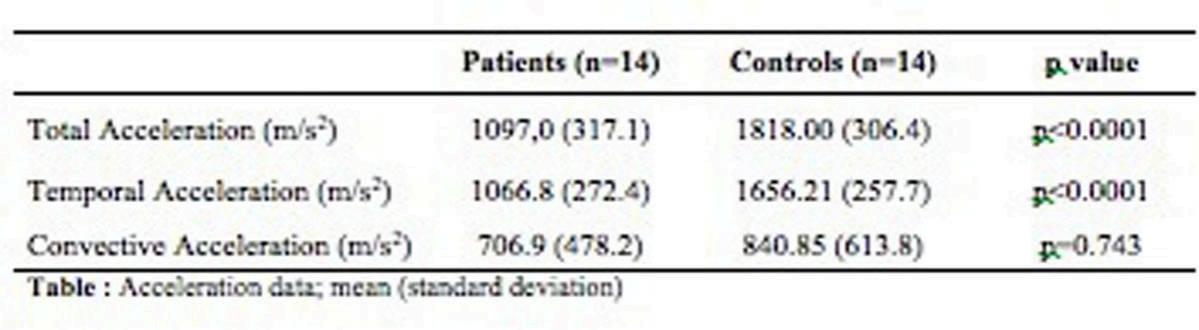
Acceleration data: mean (standard deviation)

**Figure 2:**
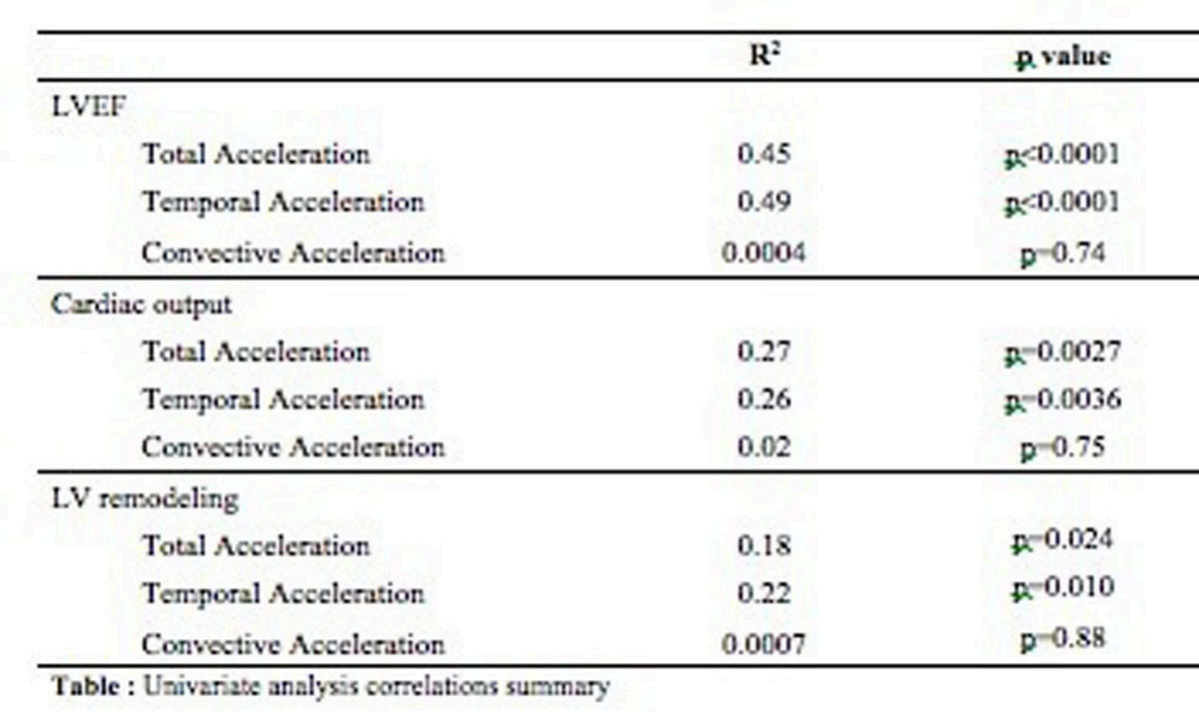
Univariate analysis correlations summary.

